# Research on the Anti-Erosion Mechanism of the Shell Surface Structure Based on Numerical Simulation

**DOI:** 10.3390/biomimetics11010062

**Published:** 2026-01-12

**Authors:** Zhenjiang Wei, Chengchun Zhang, Xiaomin Liu, Chun Shen, Meihong Gao, Jie Li, Zhengyang Wu, Meihui Zhu

**Affiliations:** 1 Department of Mechanical and Electronic Engineering, Heze University, Heze 274015, China; zhenjiangwei@hezeu.edu.cn (Z.W.); liuxiaomin@hezeu.edu.cn (X.L.); gaomeihong@hezeu.edu.cn (M.G.); lijie@hezeu.edu.cn (J.L.); 2 National Key Laboratory of Automotive Chassis Integration and Bionics, Jilin University, Changchun 130022, China; shench@jlu.edu.cn; 3 Institute of International Education, New Era University College, Kajang 43000, Malaysia; 4 College of Mechanical & Electrical Engineering, Henan Agricultural University, Zhengzhou 450002, China; zywu24@henau.edu.cn

**Keywords:** biological surface, erosion, liquid-solid two-phase flow, particle trajectory

## Abstract

This paper introduces a biological surface that is resistant to erosion under liquid–solid two-phase flow. Numerical simulations are used to study the erosion of smooth and ribbed shells by particles. The results show that when the flow direction is perpendicular to the direction of the shell ribs, the total erosion rate of the ribbed shell is 29.08% lower than that of the smooth shell, and the impact velocity of particles with a diameter of 0.5 mm on the ribbed shell is 15.91% lower than that on the smooth shell. This phenomenon occurs because a low-velocity flow field is formed in the grooves of the ribbed shell, which causes the particles to decelerate for some time before impacting the shell. This ribbed structure may provide design ideas for equipment that is susceptible to erosion.

## 1. Introduction

Erosion wear occurs in transportation equipment used in the gas and oil production industry that involves slurry containing solid particles, which may result in equipment degradation [1,2,3,4,5]. To reduce the erosion of multiphase flow equipment caused by the impact of solid particles on the surfaces of materials, a common approach is to coat wear-resistant materials on the equipment surface [6,7,8]. Although coatings can improve the anti-erosion performance at some impact angles to a certain extent, very serious erosion will still occur at local locations [9,10].

Biological surfaces are the active interfaces between subjects and the environment, and are evolving to a high state of intelligent functionality. By adopting these functional surfaces, many problems have been solved in engineering practice [11,12,13,14,15]. For example, Parra et al. designed a 3D falcon-inspired prototype with oscillating feathers and demonstrated, via experiments and CFD, that the resulting biomimetic vortex generators can improve the wake and aerodynamic performance of horizontal-axis wind turbines [11]; Dai et al. developed and optimized a bionic ornithopter whose wing structure enhances flapping flight efficiency [12]. At the fluid–structure interface, Wei et al. showed that an adherent gas layer at a solid wall can actively manipulate bubble-collapse patterns and thereby control cavitation–wall interactions [13], while Zhang et al. and Li et al. reported coupling structures and surface chemistries inspired by ladybird elytra and natural icephobic systems that provide robust waterproofing and anti-icing functions in harsh environments [14,15]. For biomimetic erosion resistance, Ren et al. found that the surface morphology and multilayered structure of desert lizards contribute to enhancing the erosion resistance of biological materials. They elucidated the anti-erosion mechanism of desert lizards based on stress wave theory [16,17,18]. Han et al. reported that the protrusions, grooves, pits, and curvatures on the scorpion exoskeleton exhibit excellent erosion resistance, and they elucidated the underlying mechanisms of these structural features [19,20,21,22]. Baumgartner et al. found that the most remarkable adaptation of the sandfish to its subterranean habitat lies in its epidermis, which exhibits low-friction behavior and outstanding resistance to sand abrasion [23]. Wang et al. found that the conch shell can serve as an inspiration for optimizing hydraulic valve cores, where the incorporation of biomimetic anti-erosion structures enhances their erosion resistance [4].

Here, the concept of an anti-erosion surface originated from the shapes of ocean scallops, which present a wave feature. Despite living in a muddy sand environment that is susceptible to erosion, they maintain a smooth and intact surface, which provides inspiration for solving erosion problems in engineering applications. Numerical simulation was used in the present research to study the kinematics and dynamics of particles to verify the erosion resistance of the shell surface.

Computational fluid dynamics (CFD) has been widely used to solve liquid–solid two-phase flow engineering problems, including the erosion caused by solid particles in fluids. Many investigators have explored the erosion model since Finnie in 1960 [24]. For instance, Meng and Ludema [25] reviewed 28 models specifically for the investigation of solid particle-wall erosion before 1995. Additionally, Derrick and Michael [26] introduced some erosion models that have been used in CFD-based analysis from 1994 to 2012. Computational fluid dynamics-discrete phase modeling (CFD-DPM) and the computational fluid dynamics-discrete element method (CFD-DEM) are two methods for the simulation of the movement of particles in turbulent flow. CFD-DPM is usually used for the numerical calculation of dilute flow, as particle-to-particle interactions are ignored in this method [27,28,29,30]. The CFD-DEM can be used to simulate the movement of particles, and the microscopic dynamic behavior of each particle can be calculated and tracked [31,32,33,34]. Based on this approach, Yasser et al. investigated the erosion behavior of 90° elbows under particles of different shapes and found that, compared with spherical particles, polyhedral particles caused the most severe erosion due to their sharp edges [33]. Sun et al. analyzed the wear characteristics of particle-laden flows impacting flat and ribbed walls under different particle incident angles, velocities, and volume fractions. The results showed that square ribs are more effective in specifically reducing the erosion rate of the grooved surface [32].

In this work, the anti-erosion mechanism of the shell surface is investigated by combining experimental preparation of biological models with numerical simulations. Real shells are cleaned, and two representative specimens are prepared: one preserves the natural ribbed morphology, while the other is filled and smoothed to obtain a shell with nearly identical overall size but without ribs. The acquired point-cloud data are processed in reverse-engineering software to generate accurate ribbed and smooth shell geometries for subsequent simulations. CFD–DEM is then employed to compute the liquid–solid two-phase flow over the shells, resolving both the continuous-phase velocity and pressure fields and the discrete particle trajectories and impact forces in order to elucidate the erosion-resistance mechanism of the ribbed surface. The remainder of this article is organized as follows. Section 2 details the experimental preparation and 3D scanning of the shell samples and the construction of the biological models. Section 3 and Section 4 describe the numerical methodologies, including the governing equations, erosion model, and simulation setup. Section 5 presents and discusses the flow-field and erosion results for smooth and ribbed shells under different flow orientations, and Section 6 concludes the paper.

## 2. The Establishment of Biological Models

### 2.1. Sample Preparation

**Figure 1:**
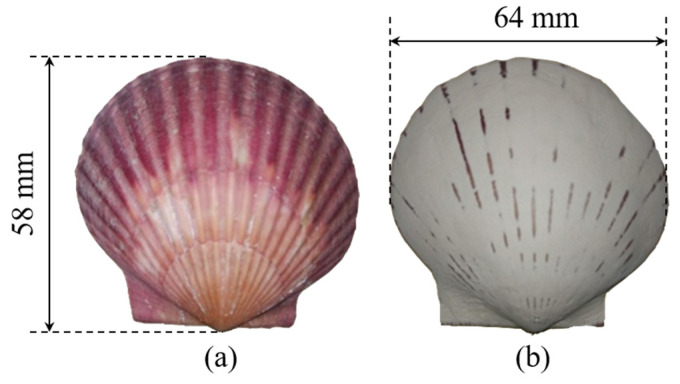
Shell samples: (**a**) ribbed shell; (**b**) smooth shell.

The surface structure of a scallop shell is complicated, and it is difficult to model directly with 3D modeling software; thus, the reverse-engineering method was adopted to generate the geometric model. Well-grown scallops (*Argopecten irradias*, living in the Bohai Sea, China) were selected and eviscerated, and two shells were obtained. First, the attachment on the scallop shell was gently suspended by a blade and then placed in an ultrasonic cleaner for 15 min. A soft brush was then used to remove the sediment and other impurities in the surface gap. The shells were rinsed 5 times with clean water and then dried. One of the shells was coated with a mixture comprising water and gypsum powder to make it smooth. The other shell was not processed. A ribbed shell and a smooth shell of the same size were prepared, whose sizes are 58 mm × 64 mm and thicknesses are 13 mm, as shown in Figure 1.

The cuticle color of the shell surface was uneven and dark, and the surface was reflective and complex in shape, with many small structures. An imaging agent (Shanghai Xinmeida Flaw Detection Material Co., Ltd., Shanghai, China) was sprayed onto the surface to endow the shell with good diffuse reflectivity and to improve the scanning data; high-quality point cloud data could be easily obtained, and small particles would not affect the scanning accuracy. First, the two shells were placed flat on the table. After the imaging agent was shaken, spraying began at a distance of 20 cm from the shells. The injection angle was slowly changed to make the spray fall evenly on the surfaces of the shells to avoid the accumulation of the reagent and the loss of surface structure details, which would increase the scanning error. After spraying, the shells were placed in a ventilated area to dry.

### 2.2. Acquisition and Processing of Shell Surface Data

**Figure 2:**
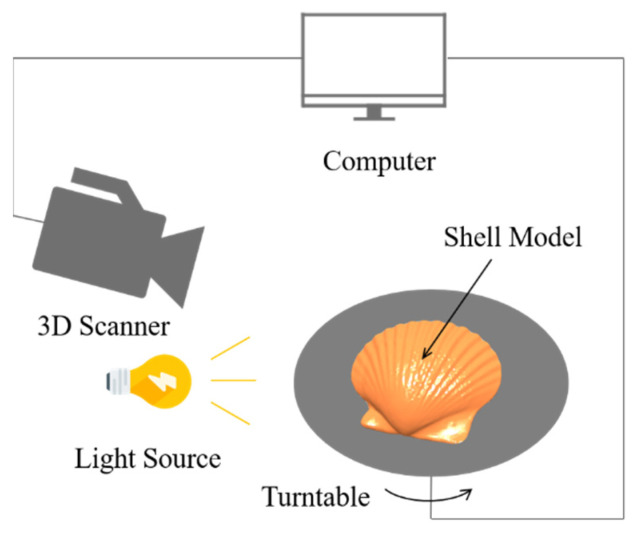
3D scanning diagram of the shell model.

**Figure 3:**
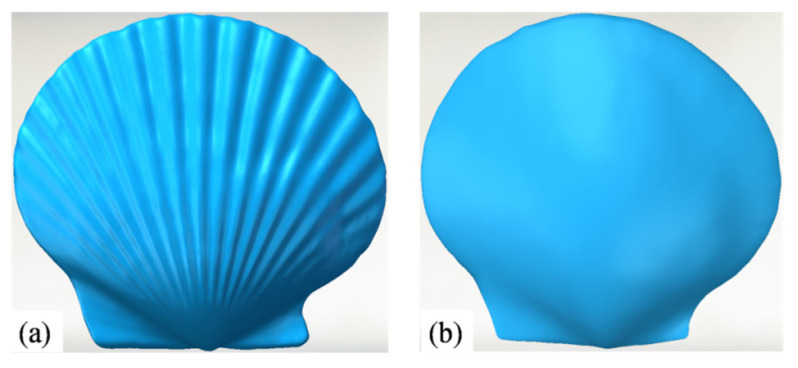
The geometric models of the scallop: (**a**) ribbed shell; (**b**) smooth shell.

Three-dimensional scanners (COMET L3D 5M, Steinbichler Optotechnik, Neubeuern, Germany) were used to obtain shell surface data, as shown in Figure 2. Each 3D scanner was equipped with an electric turntable system for the automatic positioning of the small workpiece. The cleaned shells were fixed to the turntable, the position and perspective of the 3D scanner were adjusted, the coordinate system was calibrated, and COMETplus software (version 9.6) was set up to control the slow rotation of the turntable. While the 3D scanner software could pre-process point cloud data into polygon mesh data, the obtained 3D model had some defects and high roughness. Geomagic Studio is a professional reverse-engineering processing software developed by the Geomagic Corporation in the United States, and is used to convert point cloud and polygon mesh data into accurate 3D geometric models. This software was therefore used in the present study to process and repair the point clouds, polygons, and surfaces generated from the scanned data to generate high-quality 3D models of the shell surfaces, as shown in Figure 3. Based on several representative radial profiles extracted from the reconstructed model, the shell spans a radial angle of 115 and contains 19 ribs in total. The ribs exhibit a characteristic crest-to-valley height of approximately 3.6 mm, with an average crest-to-crest spacing of 4.7 mm along the circumferential direction. The smooth shell model was generated from the same specimen by filling the grooves while preserving the overall shell size (58 mm × 64 mm in planform and 13 mm in thickness), so that the only difference between the two models is the presence of the ribbed topography.

## 3. Numerical Methodologies

The CFD-DEM coupling method was used for the numerical simulation of the liquid–solid two-phase flow over scallops. CFD was used to analyze the pressure and velocity flow fields of fluids around the scallops, and the DEM was employed to analyze the particle trajectories and impact forces on the scallops. The micro-dynamic behavior of every particle can be calculated and traced in the CFD-DEM method, although it consumes a lot of computing resources. Only the drag force was considered as the liquid–solid interactional force, and the lift and heat transfer models were not considered. The process of coupling calculation is described as follows. CFD was used to calculate the continuous-phase flow field at a certain time until convergence, and the flow-field information, such as velocity and pressure, was converted into the fluid drag force acting on the particles in the DEM via the coupling transfer function of the drag force model. The DEM was used to calculate the external forces, such as drag force and collision force, that each discrete phase particle received, and the information, such as the position and velocity of the particle in this time step, was updated. Finally, the particle information was transferred to CFD in the form of the momentum equation via the coupling function, thereby affecting the flow field.

### 3.1. Flow Model

The incompressible unsteady Navier–Stokes equations were used to solve the fluid flow. Due to their familiarity, they are not described here. To obtain an accurate solution to the Navier–Stokes equations, the turbulent flow was solved using the standard k−ε model with the standard wall functions. The model solves the fully turbulent region, does not solve the equations in the viscosity-affected inner region, and uses the wall functions to bridge the viscosity-affected region between the wall and the fully turbulent region.

(1) $$\left\{\begin{array}{l} \frac{\partial }{\partial t}\left(\rho k\right)+\frac{\partial }{\partial x_{i}}\left(\rho ku_{i}\right)=\frac{\partial }{\partial x_{j}}\left[\left(\mu +\frac{\mu_{t}}{\sigma_{k}}\right)\frac{\partial k}{\partial x_{j}}\right]+G_{k}+G_{b}-\rho \varepsilon -Y_{M}\\ \frac{\partial }{\partial t}\left(\rho \varepsilon \right)+\frac{\partial }{\partial x_{i}}\left(\rho \varepsilon u_{i}\right)=\frac{\partial }{\partial x_{j}}\left[\left(\mu +\frac{\mu_{t}}{\sigma_{\varepsilon }}\right)\frac{\partial \varepsilon }{\partial x_{j}}\right]+C_{1\varepsilon }\frac{\varepsilon }{k}\left(G_{k}+C_{3\varepsilon }G_{b}\right)-C_{2\varepsilon }\rho \frac{\varepsilon^{2}}{k} \end{array}\right.$$

where *ρ*, μ, and $\mu_{t}$ represent the fluid density, viscosity, and turbulent viscosity, respectively, *k* and ε represent the turbulent kinetic energy and dissipation rate, respectively, *G_k_* represents the generation of turbulent kinetic energy due to the mean velocity gradients, *G_b_* is the generation of turbulent kinetic energy due to buoyancy, *Y_M_* represents the contribution of the fluctuating dilatation incompressible turbulence to the overall dissipation rate, and $C_{1\varepsilon }$, $C_{2\varepsilon }$, and $C_{3\varepsilon }$ are constants. Some previous publications [35,36] provide more details about the model and parameter values.

### 3.2. Discrete Particle Motion Modeling

While the flow of fluid as a continuous phase is described on the computational cell scale, the motion of particles is modeled as a discrete phase on an individual scale and described by Newton’s laws of motion. To highlight the influence of the flow field on the movement of particles, the particles are simplified into spherical shapes. The governing equation is as follows:

(2) $$m_{p}\frac{du_{p}}{dt}=m_{p}g+F_{drag}+F_{c}+F_{vm}+F_{pg}+F_{sl}+F_{b}+F_{m}$$

where *F_drag_*, *F_c_*, *F_vm_*, *F_pg_*, *F_sl_*, *F_b_*, and *F_m_* represent the drag force, contact force, virtual mass force, pressure gradient force, Saffman lift force, Basset force, and Magnus force, respectively. To explore the influence of the flow field on the particles, gravity was not considered. A previous study [37] suggests that the drag force plays a major role in the force acting on the particles by the fluid. Under the current operating conditions, the drag force is several orders of magnitude larger than the lift force. In addition, the relatively high Froude number indicates that particle trajectories over the short interaction distance are dominated by fluid momentum transfer rather than by gravitational settling. Thus, in the present study, focus was placed only on the drag force and contact force. The drag force is used to describe the interaction between phases in multiphase flow. $F_{drag}$ is calculated as follows:

(3) $$F_{drag}=0.5C_{D}r_{g}A_{p}\left(u_{l}-u_{p}\right)\left|u_{l}-u_{p}\right|$$

where $C_{D}$ is the single-particle drag coefficient, $\rho_{g}$ is the fluid density, $A_{p}$ is a particle cross-sectional area, $u_{l}$ is the fluid velocity, and $u_{p}$ is the particle velocity. The contact force describes the particle-particle and particle-wall contact collision behaviors.

When a particle enters a turbulent flow, it tends to follow the turbulent flow. This interaction causes the lateral dispersion of the particles. The dispersion of particles caused by turbulence in the fluid phase is commonly obtained using the discrete random walk (DRW) approach [38]. This approach explains the effect of instantaneous turbulent fluctuations on the particle trajectory using a stochastic method. The particle is assumed to interact with the eddy over the smaller of the eddy lifetime ($\tau_{e}$) and the eddy crossing time ($\tau_{c}$). The eddy lifetime is defined as:

(4) $$\tau_{e}=0.3\frac{k}{\varepsilon }$$

where *k* is turbulent kinetic energy, and ε is the dissipation rate. The particle eddy crossing time is defined as:

(5) $$\tau_{c}=-\tau \ln\left[1-\left(\frac{l_{e}}{\tau \left|u-u_{p}\right|}\right)\right]$$

where τ is the particle relaxation time, *l_e_* is the eddy length scale, and $\left|u-u_{p}\right|$ is the magnitude of relative particle velocity.

The Hertz–Mindlin (no-slip) contact model [39] was used for particle-to-wall contact and particle-to-particle contact. When the particles collide with other particles or the wall, the colliding objects are deformed, resulting in normal overlap $\delta_{n}$ and tangential displacement $\delta_{t}$.The normal force, *F_n_*, is defined as:

(6) $$F_{n}=\frac{4}{3}E^{*}\sqrt{R^{*}}\delta_{n}^{\frac{3}{2}}$$

where the equivalent Young’ Modulus E*, the equivalent radius R* are given by:

(7) $$\left\{\begin{array}{l} \frac{1}{E^{*}}=\frac{\left(1-\nu_{i}^{2}\right)}{E_{i}}+\frac{\left(1-\nu_{j}^{2}\right)}{E_{j}}\\ \frac{1}{R^{*}}=\frac{1}{R_{i}}+\frac{1}{R_{j}} \end{array}\right.$$

with $E_{i}$, $\nu_{i}$, $R_{i}$, and $E_{j}$, $\nu_{j}$, $R_{j}$, being the Young’ Modulus, Poisson ratio, and Radius of each sphere in contact. If the radius of one particle is set equal to infinity, the model reduces to a particle–wall collision. Additionally, there is a damping force, $F_{n}^{d}$, given by:

(8) $$\left\{\begin{array}{l} F_{n}^{d}=-2\sqrt{\frac{5}{6}}\beta \sqrt{S_{n}m^{*}}u_{n}^{\vec{rel}}\\ m^{*}={\left(\frac{1}{m_{i}}+\frac{1}{m_{j}}\right)}^{-1}\\ \beta =\frac{-\ln e}{\sqrt{\ln^{2}e+\pi^{2}}}\\ S_{n}=2E^{*}\sqrt{R^{*}\delta_{n}} \end{array}\right.$$

where $m^{*}$ is the equivalent mass, $u_{n}^{\vec{rel}}$ is the normal component of the relative velocity, $S_{n}$ is the normal stiffness and β is a constant related to the coefficient of restitution *e*.

The tangential force *F_t_* is a function of the tangential overlap *δ_t_* and the tangential stiffness *S_t_*, defined as:

(9) $$\left\{\begin{array}{l} F_{t}=-S_{t}\delta_{t}\\ S_{t}=8G^{*}\sqrt{R^{*}\delta_{n}} \end{array}\right.$$

where $G^{*}$ is the equivalent shear modulus. Also, there is a damping force $F_{t}^{d}$ given by:

(10) $$F_{t}^{d}=-2\sqrt{\frac{5}{6}}\beta \sqrt{S_{t}m^{*}}u_{t}^{\vec{rel}}$$

where $u_{n}^{\vec{rel}}$ is the tangential component of the relative velocity. The tangential force is limited by Coulomb friction *μ_s_F_n_*, where *μ_s_* is the coefficient of static friction.

### 3.3. Erosion Modeling

Given its mechanism and large number of factors, particle erosion is a very complicated process. In engineering applications, the Oka and DNV erosion models are commonly used; however, both were primarily developed for ductile metallic alloys subjected to sand-particle erosion. The Oka and DNV models require additional empirical coefficients that strongly depend on detailed material characterization—such as tensile strength, Vickers hardness, heat-treatment condition, and ductile–brittle transition behavior—none of which are available for the natural shell materials used in this study. The aim of the present work is to compare the relative erosion resistance of biomimetic geometries with different orientations. The Archard model is well-suited for such comparative and mechanistic analyses because it directly relates local wear to the normal impact force, sliding distance, and contact load, and it is less sensitive to missing material parameters.

In this work, it was considered that the amount of material removed from the surface was proportional to the frictional work done by particles moving over the surface. Therefore, the Hertz–Mindlin model with the Archard wear model was adopted to estimate the wear depth for the geometric surfaces, as follows:

(11) $$\begin{array}{l} Q=WF_{n}d_{t}\\ wear\;depth=\frac{Q}{A} \end{array}$$

where *Q* is the volume of material removed, *d_t_* is the tangential distance moved, *W* represents a wear constant related to the material hardness, whose value is 6 × 10^−11^ pa^−1^, *F_n_* is the normal contact force by an individual particle, and *A* represents the average cross-sectional area of material removed. Since this is a simplified erosion model, the simulation results can be used to compare the erosion intensity of different locations.

## 4. Details of the Simulations

### 4.1. Description of the Geometry

**Figure 4:**
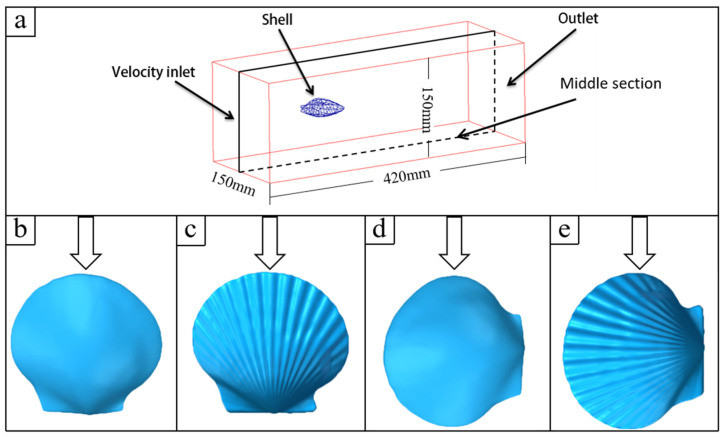
(**a**) CFD calculation domain. (**b**) Smooth shell model with vertical flow. (**c**) Ribbed shell model with vertical flow. (**d**) Smooth shell model with parallel flow. (**e**) Ribbed shell model with parallel flow.

The fluid calculation domain was a cuboid with dimensions of 420 × 150 × 150 mm^3^, as shown in Figure 4a. To obtain the fully developed flow, the models of the two sets of shells obtained by scanning were placed at the center of the section at a distance of 130 mm from the velocity inlet. The centerlines of the ribbed and smooth shells were, respectively, perpendicular and parallel to the incoming flow direction, and 4 models were established in total, as shown in Figure 4b–e, in which the arrows represent the flow direction.

### 4.2. Computational Mesh

**Figure 5:**
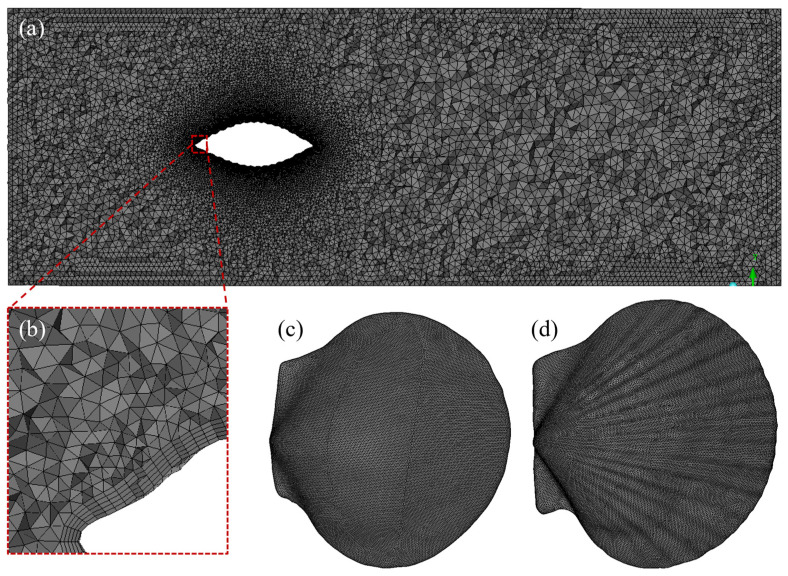
Numerical computation grid. (**a**) Computational domain, (**b**) the enlarged image in (**a**), (**c**) smooth shell surface, and (**d**) ribbed shell surface.

An unstructured tetrahedral mesh was used for the calculation, and the meshes of the shell surfaces are presented in Figure 5. The mesh was appropriately densified to ensure the quality of the mesh. The initial height of the boundary layer on the surface was 0.02 mm with a growth rate of 1.04×. There were 5 layers in total, and the wall-normal non-dimensional distance (*y*^+^) was 32. Due to the differences in the geometric models, the number of mesh elements was slightly different.

### 4.3. Simulation Modeling

Numerical calculations were performed via the CFD-DEM. The CFD code in ANSYS FLUENT software (2025 R1) was employed to model the continuous-phase flow. A pressure-based, transient-in-time solver was adopted. The temperature was set to 293 K, and the influence of temperature on the simulation experiment was not considered. The pressure-velocity coupling was used as a solution method, whose scheme is Phase Coupled SIMPLE. Because the flow velocity is slow and there is no vortex and backflow, the first-order upwind was used for discretization. Least Squares Cell-Based was used for gradient discretization. The modeling of discrete particle motion was accomplished by the DEM code EDEM. Individual particle motion characteristics were revealed, and particle-particle and particle-wall collisions were tracked. To ensure that the simulation remains consistent with realistic operating conditions and to avoid flow separation caused by excessively high velocities. The inlet was set to the velocity-inlet boundary condition, the velocity was 5 m/s, and the turbulence intensity was 5%. The outlet was set to the outflow. The properties of the particles and the water carrier fluid, as well as the boundary conditions, are summarized in Table 1.

The particle factory was used to release particles. While keeping the mass flow of two particles consistent, a dynamic and unlimited number of particles was adopted to produce 1000 particles per second for larger particles (*d* = 1 mm) and 8000 particles per second for smaller particles (*d* = 0.5 mm). The release position was random, and the velocity and direction were continuous, namely 5 m/s. The corresponding particle mass flow rate is 0.00278 kg/s, and the solid volume fraction is 0.00107. This setup reduces the influence of particle–particle collisions on material erosion and allows us to focus on the erosion mechanisms of particles impacting the shell wall surface. The calculation time for one step in the flow field is 4 × 10^−5^ s; there are a total of 12,500 time steps, and the maximum number of iterations per step is 200. In each calculation step of the flow field, the particle data is updated 4 times during the calculation step, and the time step for calculating the particle is 1 × 10^−5^ s.

### 4.4. Mesh Independence

The accuracy of the mesh has an important influence on the calculation results, and the density and quality of the mesh must be determined via a mesh independence analysis. In general, when performing a mesh independence analysis, at least three sets of calculation experiments are performed to explain the effect of the grid density on the results. Wilson [40] recommended that the number of nodes on each side of the grid increase by $\sqrt{2}$ times. Three different mesh numbers were, respectively, adopted for the smooth and ribbed shell models, as shown in Table 2 and Table 3. In the two cases in which the incoming flow velocity was 5 m/s, three kinds of mesh were used to calculate the shell erosion rate within 0.5 s. When the number of meshes reached over 5 million, the error rate was within 3%; thus, the mesh number of 5,438,245 was adopted.

### 4.5. Experimental Validation

To evaluate the accuracy of the selected numerical model, a classical right-angle pipe elbow was used as the test specimen for erosion experiments. The elbow had an internal diameter of 26 cm and a bending radius ratio of 1.5. Because direct erosion tests typically require an extended period and distinct erosion patterns are difficult to develop within a short timescale, a mixed coating composed of latex paint and gypsum powder at a mass ratio of 2:1 was applied to the wall surface to enable rapid visualization of erosion features.

**Figure 6:**
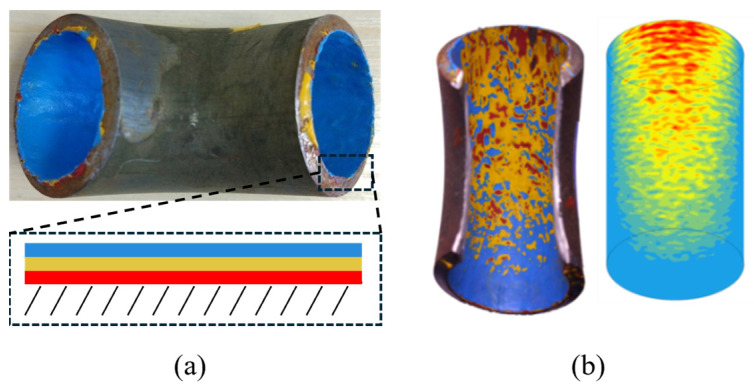
Verification of Pipeline Erosion. (**a**) Experimental pipeline; the inset shows the inner wall of the elbow coated sequentially from inside to outside with red, yellow, and blue layers; (**b**) Comparison between experimental observations and numerical simulation, where the left panel corresponds to the experiment and the right panel shows the simulation results.

In the experiment, red, yellow, and blue coatings were sequentially applied to the inner wall of the pipe elbow, as illustrated in Figure 6. The experimental conditions were consistent with those described in the previous sections. The experimentally observed erosion patterns and the simulated results are shown correspondingly. The comparison indicates that the experimental and numerical results agree well, demonstrating that the selected numerical model can reliably reproduce the erosion phenomena.

## 5. Results and Discussion

### 5.1. Erosion Analysis

**Figure 7:**
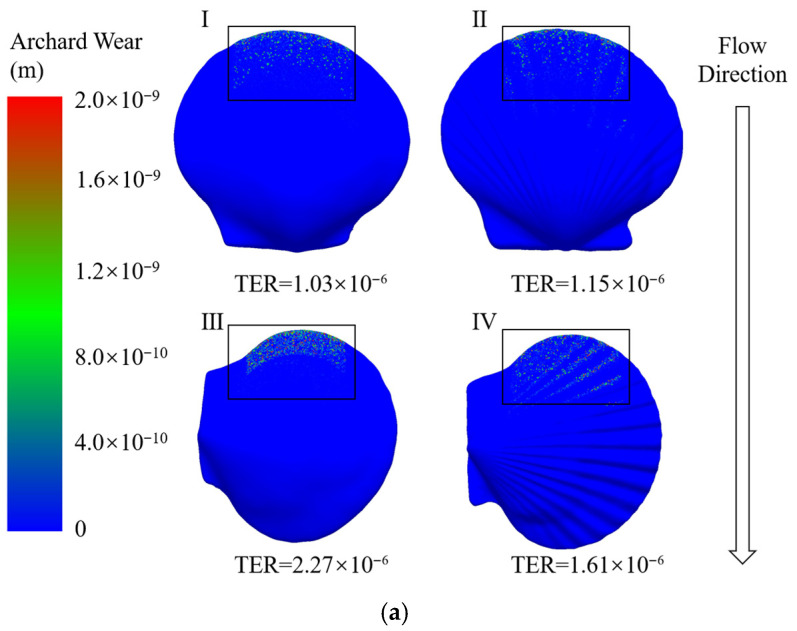

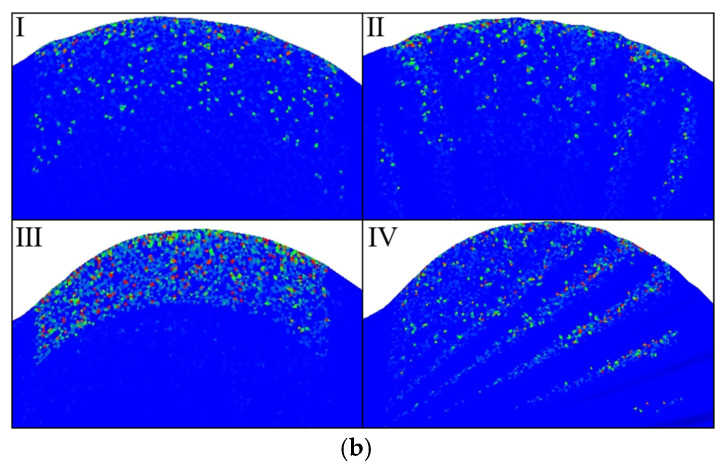
The distributions of the erosion rates caused by large particles (1 mm) and small particles (0.5 mm). The zoomed-in views of (**b**) correspond to I, II, III, and IV of (**a**), respectively.

According to the Archard wear model, the erosion rate is defined as the depth of the wall material per unit area that is reduced by the impact of particles. The respective erosion contours formed by the smooth shell and ribbed shell in the parallel and perpendicular flow directions are shown in Figure 7. Erosion wear only occurred on the front part of the shell in all simulation calculations. An obvious erosion boundary was produced on the smooth shell. Because the effect of gravity was not considered, the direction of movement of the particle did not change before colliding with the shell, and the corresponding erosion contours were formed according to the relative geometric relationship of the shells. In contrast, when the direction was vertical, erosion mostly occurred on the top of the ribs; almost no erosion occurred in the grooves between the ribs.

The depth of the eroded material was considered as the erosion rate in the simulation calculation. The total erosion rate (TER) is defined as the integral of the erosion rate on the surface area, as shown in Figure 7. The results indicate that when the flow direction is perpendicular to the ribbed orientation of the shell, the total erosion rate (TER) is much higher than in the case where the flow direction is parallel to the ribs. This difference arises from the distinct particle impact locations on the shell surface, which in turn lead to different incident angles during particle–wall collisions. Variations in incident angle cause corresponding differences in the normal impact force. For particles with the same impact velocity, the model placed in the perpendicular orientation experiences a larger incident angle and thus a higher normal impact force. According to the erosion model, the normal impact force is the dominant contributor to erosion wear, and therefore, a significantly higher TER is observed in the perpendicular configuration. When the direction of water flow was parallel to the shell, the TER of the ribbed shell was slightly greater than that of the smooth shell. However, when the direction was perpendicular to the shell, the TER of the ribbed shell was much lower than that of the smooth shell, exhibiting a reduction of 29.08%. This phenomenon is subsequently explained in detail.

The erosion rate of the material is affected by many factors. In this simulation experiment, there is little difference in other factors. The main consideration is the effect of the flow field on the particle velocity and trajectory.

### 5.2. Velocity Analysis

**Figure 8:**
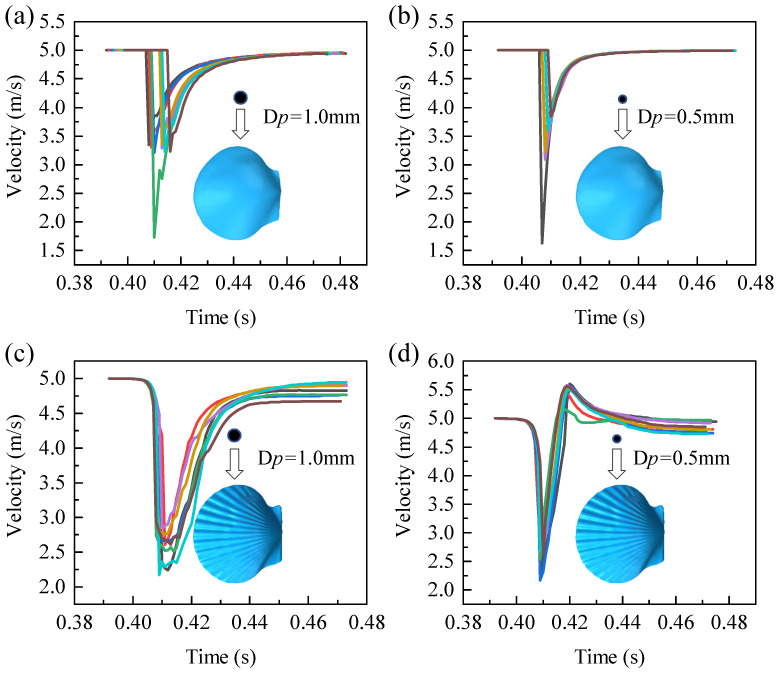
Velocity–time curves of particles. (**a**) The velocity–time curves of particles with a diameter of 1 mm impacting a smooth shell. (**b**) The velocity–time curves of particles with a diameter of 0.5 mm impacting a smooth shell. (**c**) The velocity–time curves of particles with a diameter of 1 mm impacting a ribbed shell. (**d**) The velocity–time curves of particles with a diameter of 0.5 mm impacting a ribbed shell. (Each color represents a different particle).

When the direction of the shell ribs was perpendicular to the direction of the water flow, the ribbed shell exhibited better erosion resistance than the smooth shell. In liquid–solid two-phase flow, the impact velocity of particles has significance for the erosion rate. In liquid–solid two-phase flows, the rate of material erosion is strongly governed by the velocity at which particles impact the wall. To reduce accidental errors, eight particles of each size are selected for velocity analysis, as shown in Figure 8. Under the action of fluid flow, the velocity of the particles underwent four stages, namely an initial plateau period, an impact deceleration period, a recovery period, and an end plateau period. In the smooth shell model, the average time (0.0154 s) required for small particles from deceleration to acceleration to the original speed was shorter than that for large particles (0.0400 s). Because particles with a smaller diameter have a smaller Stokes number, which is a dimensionless number that reflects the following characteristics of the particles, the smaller the value, the better the following characteristics, and the shorter the amount of time it takes for the particles to change with the fluid velocity.

In the obtained velocity curves, two points are particularly noticeable: (1) from the initial stable period to the impact deceleration period, the change in velocity formed smooth curves in the ribbed shell model and non-smooth curves in the smooth shell model; (2) when particles with a diameter of 0.5 mm hit the ribbed shell wall, these particles appeared to exceed the initial fluid velocity after the recovery period, which did not occur in the smooth shell model. These two phenomena are subsequently explained in detail.

### 5.3. Flow-Field Analysis

**Figure 9:**
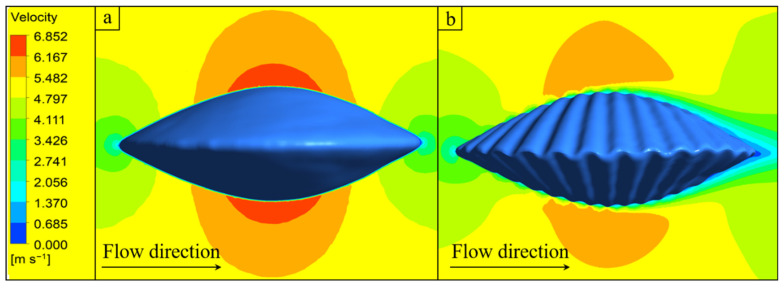
(**a**) Contours of particle velocity on the smooth shell. (**b**) Contours of particle velocity on the ribbed shell.

Particles are mainly subjected to drag in the flow field, and the velocity distribution of the flow field has an important influence on the movement and erosion of the particles. Duarte et al. [41] investigated the effects of different sand particle concentrations on the erosion of an elbow pipe by using the one-, two-, and four-way coupling methods. They found that, with the increase in the particle concentration, the movement of particles and the interaction between particles have non-negligible effects on the flow field. In the present numerical simulation calculation, because the particles all moved in one direction, the interaction between the particles was not obvious, and the two-way coupling method that considers the influence of particle movement on the fluid was adopted. To reveal the velocity distribution more clearly, a section was created that was parallel to the direction of the incoming flow at the center of the computational domain, shown in Figure 4a. Figure 9 presents the contours of the velocity of the particles on the shells. Compared with the smooth shell, local low-velocity flow fields were formed in the grooves of the ribbed shell. When the particles passed through the low-speed flow fields formed by the ribs and were about to hit the wall, the velocity of the particles was found to gradually decrease, and smooth velocity curves were formed because of the drag force of the fluid. Because the smooth shell had no ribs, a low-velocity flow field similar to that of a ribbed shell is not formed, which causes the particles to hit the wall without sufficient deceleration, thereby creating a sudden change in particle velocity, as shown in Figure 8. Xu et al. [42] investigated rectangular, V-shaped, and elliptical micro-grooves under particle-laden impinging flows and reported that the rib-like biomimetic structures promote the formation of local low-velocity regions and effectively shield the underlying surface from direct particle impact. This observation is consistent with the phenomena identified in the present study.

### 5.4. Particle Trajectory Analysis

**Figure 10:**
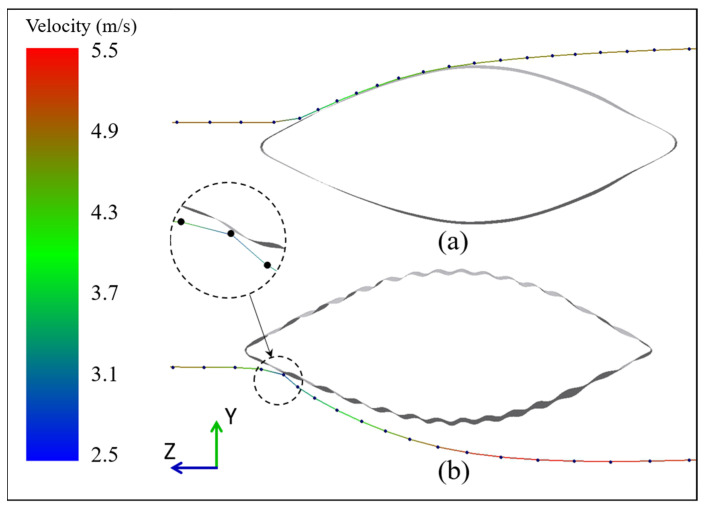
(**a**) The trajectory of a particle on the smooth shell wall. (**b**) The trajectory of a particle on the ribbed shell wall. (The color of the trajectory represents the magnitude of the particle speed).

It is of great significance to study the trajectory of particle movement to analyze wall erosion phenomena. In the numerical simulation calculation, although the relative positions of the particles colliding with the wall were different, the motion laws of the particles were similar. It was difficult to observe the details of particle motion, as there were thousands of particles in the computational domain simultaneously. Therefore, in the middle section shown in Figure 4a, a slice with a thickness of 3 mm was created to limit the number of particles. In each numerical simulation, a particle (*d* = 0.5 mm) with a typical trajectory was selected for analysis, as shown in Figure 10. The particle always moved downstream along the windward wall after colliding with the smooth shell model. In contrast, in the ribbed shell model, the particle left the wall immediately after hitting it and maintained a certain distance from the wall during movement. It is this particle behavior that reduced the impact between the particles and the ribbed shell wall, thereby reducing the erosion rate.

**Figure 11:**
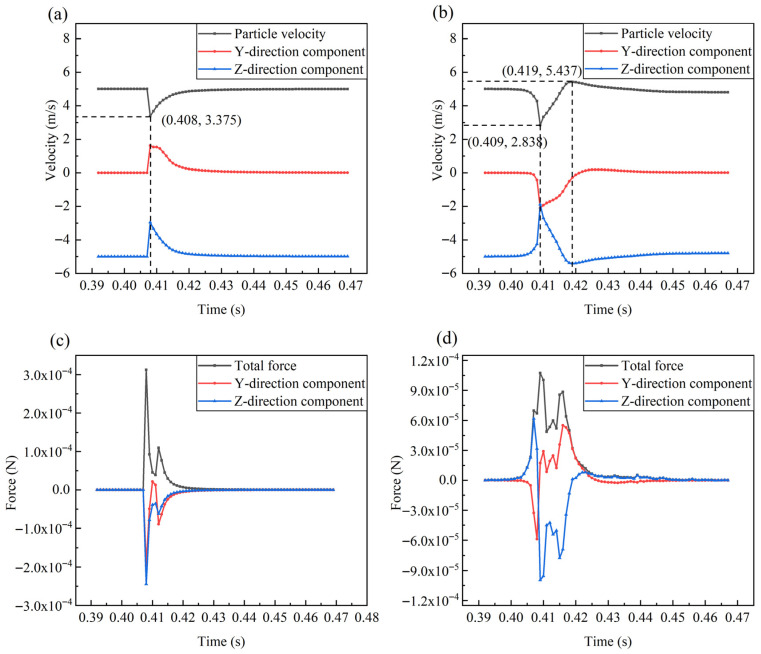
The force and velocity curves of a particle with a diameter of 0.5 mm. (**a**) The velocity curve of a single particle hitting the smooth shell. (**b**) The velocity curve of a single particle hitting the ribbed shell. (**c**) The force curve of a single particle hitting the smooth shell. (**d**) The force curve of a single particle hitting the ribbed shell.

**Figure 12:**
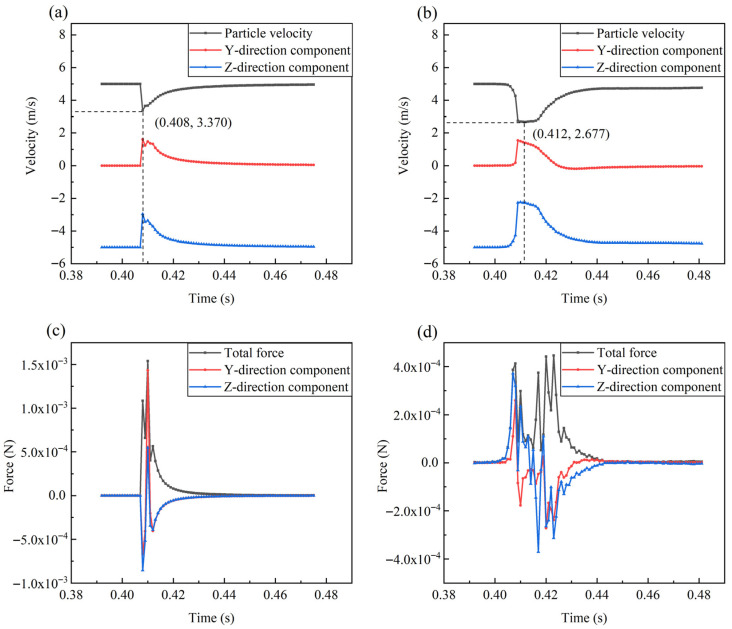
The force and velocity curves of a particle with a diameter of 1.0 mm. (**a**) The velocity curve of a single particle hitting the smooth shell. (**b**) The velocity curve of a single particle hitting the ribbed shell. (**c**) The force curve of a single particle hitting the smooth shell. (**d**) The force curve of a single particle hitting the ribbed shell.

The velocity and force of the particle at each moment presented in Figure 10 were analyzed, as exhibited in Figure 11 and Figure 12. In front of the impact wall, compared to the smooth shell simulation, the particle that hit the ribbed shell took a long time to decelerate. This is because the nearby fluid velocity was less than the particle velocity, and the fluid had an obstructive effect on the particle. A lower impact velocity (2.838 m/s) was produced, which was 15.91% less than that of the particle in the smooth shell model (3.375 m/s), and the impact force at this moment was reduced to 1/3. A lower impact velocity and impact force are of great significance to the erosion resistance of ribbed shells. A particle that collides with the wall will have a velocity slower than the fluid velocity for a certain period. At this time, the effect of the fluid on the particle will appear to be a push that accelerates the particle. Due to the action of the fluid, the velocity of the particle will gradually increase. According to Equation (3), when the velocity difference between the fluid and a particle decreases, the driving force of the particle also decreases; thus, the total force of the particle also decreases. Subsequently, the particle enters the higher-velocity flow field, and the velocity difference increases, so the force becomes larger. As the particle accelerates, the velocity difference decreases, and the total force also decreases. In this process, the speed of the particle will continue to increase, and the resultant force is constantly changing. After this, the velocity difference between the particle and the fluid will continue to decrease, and the total force experienced by the particle will also decrease, eventually tending to 0. This elicits the question of why the maximum velocity of particles can exceed the initial velocity in the ribbed shell model, but not in the smooth shell model. After colliding with the wall, the particle in the smooth shell simulation was found to achieve a lower velocity and had lower kinetic energy. Then, in the process of moving along the wall of the smooth shell, the energy transferred by the fluid to the particles was not completely converted into the kinetic energy of the particle due to the obstruction of the wall. Due to these reasons, the velocity of the particle leaving the wall of the smooth shell model was 4.653 m/s, whereas the particle velocity for the ribbed shell model was 5.045 m/s. The particles in the two models accelerated in the region of the highest-speed flow field. Although the fluid of the smooth shell model had a greater speed in this region, the particle left the high-velocity region without completely accelerating. In contrast, the particle in the ribbed shell model briefly accelerated to a speed of 5.437 m/s. Therefore, to reduce erosion, it is important that the particle maintains a considerable relative distance from the wall after it hits, and that a considerable velocity emerges for the particle to escape the wall. Chen et al. [43] demonstrated that the groove–particle interaction mechanism is primarily governed by the ability of surface textures to modify the impact angle, reduce the particle kinetic energy upon collision, and promote particle rebound. The flow-field evolution and impact–velocity distribution obtained in our simulations exhibit the same mechanisms, confirming that the ribbed biomimetic surface functions through similar hydrodynamic effects.

## 6. Conclusions

This article investigated the erosion distribution law of particles impacting shells and the mechanism of particle movement in liquid–solid two-phase flow. The CFD-DEM was used for the numerical calculation of flow fields and forces. The conclusions can be drawn as follows:

1. When the direction of the incoming flow was perpendicular to the direction of the shell rib, the total erosion rate of the ribbed shell was 29.08% lower than that of the smooth shell, thereby exhibiting good erosion resistance.
2. The low-velocity flow field that can be formed in the grooved area of the ribbed shell can buffer the particles about to hit the shell wall. The velocity of particles hitting the ribbed shell was 15.91% lower than that of particles hitting the smooth shell, thereby reducing the erosion of the ribbed shell.
3. After hitting the smooth shell wall, particles were found to move downstream along the wall. In contrast, after hitting the ribbed shell, particles were found to move at a certain distance from the wall, and under the action of the high-velocity flow field, the particles could escape the wall at a higher velocity.

However, most of the particles were found to collide with the ribs of the ribbed shell, which bear most of the impact in liquid–solid two-phase flow. If this type of bionic structure is used in mechanical equipment that is easily eroded, the ribs should be of particular concern. M.G.

## Figures and Tables

**Table 1:** Parameters used in the CFD-DEM analysis.

Item	Fluid	Particles	Shell Surface
Density (kg/m^3^)	998.2	2650	7800
Viscosity (kg/m∙s)	1.003 × 10^−3^	/	/
Hardness	/	7 (Mons’)	HB 538
Particle diameter (mm)	/	1, 0.5	/
Shape factor	/	1.0	/
Velocity (m/s)	5	5	/
Poisson’s ratio	/	0.5	0.3
Shear modulus (Pa)	/	1 × 106	7 × 1010
Mass flow rate (kg/s)	/	2.78 × 10^−3^	/
Solid volume fraction	/	1.07 × 10^−3^%	/
Coefficient of restitution	/	0.5	0.5
Coefficient of static friction	/	0.5	0.5
Coefficient of rolling friction	/	0.01	0.01

**Table 2:** Mesh sensitivity analysis of the smooth shell simulation.

Smooth Shell	Erosion Process Time (s)	Erosion Rate (m)	Error Rate
1,869,528	0.5	1.11 × 10^−6^	5.60%
5,234,677	0.5	1.03 × 10^−6^	1.90%
14,657,095	0.5	1.05 × 10^−6^	/

**Table 3:** Mesh sensitivity analysis of the ribbed shell simulation.

Ribbed Shell	Erosion Process Time (s)	Erosion Rate (m)	Error Rate
1,942,230	0.5	1.30 × 10^−6^	12.3%
5,438,245	0.5	1.18 × 10^−6^	2.97%
15,227,086	0.5	1.15 × 10^−6^	/

## Data Availability

The original contributions presented in this study are included in the article. Further inquiries can be directed to the corresponding authors.
